# Association of Age-Related Hearing Loss with Domain-Specific Cognitive Performance in Older Adults

**DOI:** 10.3390/jcm15062322

**Published:** 2026-03-18

**Authors:** Tatiana Marques, João Castelhano, Isabel Catarina Duarte, Carla Pinto-Moura, Miguel Castelo-Branco, António Miguéis

**Affiliations:** 1 Coimbra Institute for Biomedical Imaging and Translational Research, ICNAS, University of Coimbra, 3000-548 Coimbra, Portugal; 2 Faculty of Medicine, University of Porto, Alameda Prof. Hernâni Monteiro, 4200-319 Porto, Portugal; 3 Polytechnic University of Coimbra, Rua da Misericórdia, Lagar dos Cortiços, S. Martinho do Bispo, 3045-093 Coimbra, Portugal; 4 Faculty of Medicine, University of Coimbra, 3000-548 Coimbra, Portugal; 5 Unidade Local de Saúde São João, 4202-451 Porto, Portugal; 6 RISE–Health, Department of Pathology, University of Porto, Alameda Prof. Hernâni Monteiro, 4200-319 Porto, Portugal

**Keywords:** age-related hearing loss, aging, clinical features, cognitive function, educational attainment

## Abstract

**Background/Objectives**: Age-related hearing loss (ARHL) is highly prevalent among older adults and has been linked to cognitive decline. However, the specific cognitive domains most vulnerable to ARHL and whether these associations exhibit lateralized effects remain unclear, which is critical for understanding and mitigating its broader impact on neurocognitive function. This study aimed to characterize the clinical profile of ARHL and examine associations between hearing thresholds and cognitive performance across domains, including the influence of educational attainment as a proxy for cognitive reserve. **Methods**: Audiometric assessments and cognitive screening using the Mini-Mental State Examination were conducted in older adults, including normal-hearing listeners (NHL, n = 31, mean age 71.4) and those with hearing loss (HL, n = 46, mean age 73.1). Associations between pure-tone averages, clinical complaints, and cognitive domains were analyzed while considering educational attainment. **Results**: HL participants exhibited a higher prevalence of tinnitus (NHL: 33.3% vs. HL: 65.2%) and slightly more frequent dizziness compared to their normal-hearing peers. Cognitive assessment revealed that decreased cognitive performance was strongly associated with hearing loss (*p* < 0.05), and this association was influenced by low educational level. Orientation was the most affected domain (*p* < 0.01), while recall and language were also significantly associated with low- and high-frequency pure-tone averages, respectively. **Conclusions**: These findings reinforce the relationship between ARHL and cognitive decline, suggesting an attentional basis whereby higher listening effort to decode the degraded auditory input may affect cognitive performance. The results also highlight the influence of educational attainment as a moderating factor.

## 1. Introduction

Given the rise in life expectancy, age-related hearing loss (ARHL) is one of the most common age-related diseases. It is expected to affect 1.2 billion older people worldwide [1]. Overall, age-related changes in the peripheral auditory system occur due to alterations in the inner ear, specifically a decrease in the number of outer hair cells at the basal part of the basilar membrane, which affects the encoding of high-frequency information [2]. Along with these inner ear alterations, related neurophysiological changes in the auditory system, including the cochlear nuclei and other midbrain structures, can lead to numerous changes in subcortical and cortical auditory representations [3].

Consequently, the functional interaction of peripheral and central age-related hearing changes is dichotomic and clinically characterized by the development of a high-frequency sloping sensorineural hearing loss (SNHL) and poor word discrimination [4]. Furthermore, the mechanisms underlying age-related auditory dysfunction may affect shared auditory and cognitive processing pathways, contributing to the emerging connection between ARHL and cognitive function. Several studies have consistently demonstrated an association between age-related hearing loss and cognitive decline, with even small decreases in hearing thresholds leading to decreased performance of executive functions and contributing to accelerated cognitive decline [5,6,7,8,9].

Potential mechanisms have been proposed for this relationship [10,11,12,13], namely the cognitive overload hypothesis, which assumes that allocation of attentional resources due to hearing age-related changes can lead to diminished availability of cognitive resources [14,15]. According to cognitive overload theory, degraded auditory input requires greater engagement of attentional and executive resources to support effortful listening. This sustained increase in listening effort is thought to give rise to cognitive overload, whereby cognitive resources are allocated to process degraded auditory input, leading to fewer resources available for other cognitive functions, such as memory and executive control [15,16]. However, previous evidence suggests that cognitive reserve may also be a mediator between hearing loss and cognitive function, assuming that older adults with higher cognitive reserve, which is directly related to more years of formal education or more mentally stimulating activities or jobs, can more efficiently counteract the effects of neuropathologic processes [16,17].

Taken together, these findings suggest that the association between age-related hearing loss and cognitive decline is likely to be multifactorial. Accordingly, we hypothesize that both cognitive overload, possibly due to increased listening effort, and cognitive reserve may be associated with interindividual variability in cognitive outcomes among hearing-impaired older adults. While increased listening effort may accelerate the depletion of cognitive resources, higher cognitive reserve may partially delay or attenuate the cognitive consequences of age-related auditory deprivation.

In addition, the specificity of this association has focused on high-frequency hearing loss, ignoring the existence of distinct types of ARHL. For example, sensory phenotype is the audiometric typical pattern of the disease, revealing a sloping high-frequency hearing loss (≥60 dB HL), while the metabolic phenotype results in increased thresholds at lower frequencies and a gradual increase at higher frequencies. Thus, analysis of audiometric patterns may provide novel insights into sensory–cognitive association in ARHL [2].

Therefore, we designed this study to explore the clinical profile of hearing-impaired older adults, including their clinical history and symptoms, and to examine whether changes in the peripheral auditory function of geriatric patients are associated with cognitive performance. In addition, we examined whether education, as an indicator of cognitive reserve, is associated with cognitive outcomes in these hearing-impaired geriatric patients.

## 2. Materials and Methods

### 2.1. Participants

A total of seventy-seven older adults (age 65–85) were recruited from the Coimbra Institute for Biomedical Imaging and Translational Research volunteers’ database, with the collaboration of a tertiary hospital and a private medical office in Coimbra. The exclusion criteria included a history of neuropsychiatric disease and external or middle ear disease, previous ear surgery, history of head trauma, or use of ototoxic medications. Enrolled participants were allocated into two groups according to their hearing status: those who have mild to moderate SNHL were included in the hearing loss (HL, n = 46) group (25 < pure-tone average (PTA) threshold ≤ 70), and age-matched normal-hearing older adults constituted the normal hearing listener (NHL, n = 31) group (PTA thresholds ≤ 25 dB HL) (see Table 1).

Following the initial assessment, a self-administered questionnaire was used to evaluate other diseases and/or symptoms related to ARHL. It includes items with no/yes responses, as well as a set of questions designed to describe pharmacological treatment, if applicable. Participants assigned to NHL and HL groups were matched for age and sex (Table 1). The mean age of normal-hearing participants was 71.4 ± 4.56 (65–80), and the mean age of the hearing-impaired participants was 73.1 ± 5.89 (65–85). Regarding education level, older adults with hearing loss had significantly fewer years of education (U = 438, *p* = 0.004). Considering the multifactorial pathogenesis of ARHL, the presence of cardiovascular and metabolic diseases was also queried. However, there were no statistically significant differences between groups, including in hypertension, diabetes mellitus, and hyperlipidemia.

All participants provided written informed consent after the protocol was approved by the Ethics Committee of the Faculty of Medicine of the University of Coimbra (approval CE-074/2020).

### 2.2. Hearing Assessment

The participants underwent a standardized audiometric evaluation that included otoscopy, which was performed under endoscopic visualization when the eardrum morphology was uncertain, and pure-tone audiometry using an Interacoustics AD629 audiometer (Interacoustics A/S, Middelfart, Denmark) in a soundproof booth. Hearing thresholds for air conduction pure tone audiometry were obtained at frequencies of 0.25, 0.5, 1, 2, 4, 6, and 8 kHz using TDH39 headphones (Telephonics Corporation, Farmingdale, NY, USA), while bone conduction was tested with a bone vibrator at 0.25, 0.5, 1, 2, and 4 kHz to confirm the air–bone gap ≤ 10 dB HL, according to diagnostic criteria for SNHL [18]. Stimuli were warble tones. The hearing sensitivity was expressed as the average hearing thresholds at 0.5, 1, 2, and 4 kHz for each ear [19]. The pure-tone average (PTA) from both ears was calculated. Hearing was classified according to the WHO (1991) criteria: PTAs ≤ 25 dB HL were considered normal hearing, PTAs between 26 and 40 dB HL indicated mild hearing loss, and PTAs from 41 to 70 dB HL indicated moderate hearing loss [20]. Bilaterally symmetrical hearing was considered in the presence of interaural asymmetry lower than 15 dB HL in two consecutive frequencies [21].

### 2.3. Cognitive Assessment

After measuring audiometric thresholds, a cognitive screening was performed using the Mini-Mental State Examination (MMSE), which took around 10 min for each participant [22]. A Portuguese version translated and validated by Guerreiro et al. (1994) was used in this study [23]. The scale is an instrument designed to provide healthcare professionals with a reliable tool for screening the cognitive state. The instrument was used to evaluate orientation (10 points), registration (3 points), attention and calculation (5 points), recall (3 points), language (8 points), and visuospatial skills (1 point), with a total score of 30. According to education level and illiteracy, the MMSE determines the cognitive status as normal, mild cognitive impairment, or dementia [24].

### 2.4. Statistical Analysis

All statistical analysis was conducted using SPSS software (version 28.0, SPSS Inc.). The ꭓ2 test was used to compare categorical variables among groups, while the Mann–Whitney U test was applied to continuous variables. Spearman’s correlation was performed to evaluate the correlation analysis among MMSE scores, bilateral PTA, low-frequency, and high-frequency thresholds.

## 3. Results

### 3.1. Hearing Assessment

**Figure 1:**
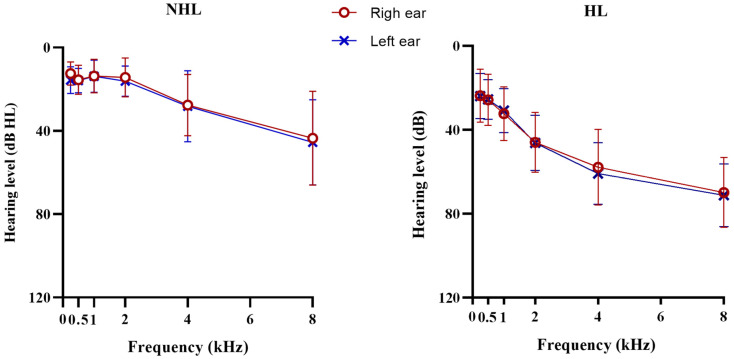
Audiograms of normal-hearing listener (NHL) and hearing loss (HL) groups.

As expected, the PTAs of hearing-impaired older adults were significantly higher compared to their normal-hearing peers (HL/NHL mean ± SD = 40.61 ± 8.35/18.15 ± 4.96), as shown in Table 2. According to the WHO classification of hearing, 22 (47.8%) participants from the HL group showed mild hearing loss, and 24 (57.2%) moderate hearing loss. As previously mentioned, there are multiple etiologies and consequently different audiometric patterns of ARHL, namely metabolic and sensory phenotypes. Therefore, to explore frequency-specific effects and better characterize audiometric configuration, PTA analyses were performed separately for low (0.25 to 1 kHz) and high (2 to 8 kHz) frequencies. A PTA of 27 (±8.88) dB HL was found for low frequencies in hearing-impaired older adults, while for high frequencies, it was 58.67 (±11.38) dB HL. See detailed PTAs by ear in Figure 1. As expected, males showed worse hearing thresholds for both frequency ranges (low-frequency PTAs: U = 125.5, *p* < 0.001; high-frequency PTAs: U = 54.5, *p* < 0.001). For the NHL group, PTA was within normal limits; however, high-frequency thresholds were slightly elevated compared to low frequencies, suggesting a mild sloping configuration.

Other clinical features commonly associated with ARHL are tinnitus, which differed significantly between groups (ꭓ2 = 7.404, *p* = 0.007), with hearing-impaired older adults reporting more frequent complaints of tinnitus (NHL: 33.3%; HL: 65.2%), as expected. Additionally, the prevalence of dizziness was slightly higher in the HL group (NHL (n, %): 5, 16.7%; HL (n, %): 10, 21.7%), although this difference was not statistically significant (ꭓ2 = 0.295, *p* = 0.770). All patients with dizziness describe brief episodes of a false sensation of rotation that is associated with head movement.

### 3.2. Cognitive Assessment

Regarding cognitive screening and the impact of education level on MMSE interpretation, participants were stratified into two subgroups based on their years of education: those with no education or less than 11 years (≤11 years) and those with higher education (>11 years). The mean score of the MMSE for the low-education NHL group (n = 14, male/female: 8/6) was 27.64 ± 1.45, while for the HL group (n = 33; male/female: 18/15), it was 26.28 ± 3.20. For the higher-education subgroups, hearing-impaired older adults (n = 13; male/female: 6/7) had similar scores to their normal-hearing peers (n = 17; male/female: 9/8). The characteristics of each subgroup are presented in Table 3, including detailed information on cognitive domain scores.

### 3.3. Lower Orientation Scores Are Associated with Higher PTA Thresholds

**Figure 2:**
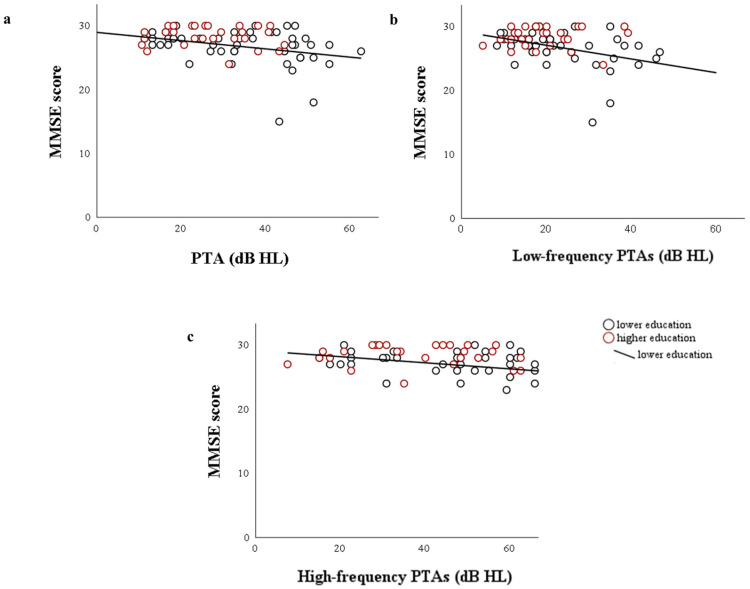
Scatterplots show the relationship between MMSE total score and (**a**) bilateral pure-tone average (PTA), (**b**) low-frequency bilateral PTAs, and (**c**) high-frequency bilateral PTAs, shown separately for both education subgroups. Linear trend lines are shown for illustrative purposes only.

Spearman’s correlation analyses were performed on the overall sample to examine the relationship between the MMSE total score, MMSE cognitive domain individual scores, bilateral PTA, and low- and high-frequency thresholds. The correlation analysis showed no significant correlation in the higher education subgroup between hearing and cognitive domains (see Figure 2). However, a significant negative relationship was observed between the total score of the MMSE and bilateral PTA (Rho = −0.325; *p* = 0.027), as well as between low-frequency PTA and the total score of the MMSE (Rho = −0.417; *p* < 0.004) in the lower education subgroup. Moreover, a marginally significant correlation was found between high-frequency PTA and the total score of MMSE (Rho = −0.283; *p* = 0.057).

Additional correlations were examined between hearing measures and cognitive domains, revealing moderate correlations between bilateral PTA and the orientation domain (Rho = −0.465, *p* = 0.001), high-frequency PTAs and orientation (Rho = −0.447, *p* = 0.002), low-frequency PTAs and orientation (Rho = −0.468, *p* < 0.001), as well as a weak correlation between low-frequency thresholds and recall domain (Rho = −0.292, *p* = 0.046) in the lower education subgroup. Moreover, this education subgroup exhibited a marginally significant correlation between high-frequency PTA and language (Rho = −0.286, *p* = 0.052). No correlations were found for the higher education subgroup.

## 4. Discussion

Despite earlier findings of an association between hearing loss and cognitive status, the lack of consensus on the clinical definition of ARHL led us to establish a clinical profile and to explore the associations between hearing thresholds and cognitive performance.

Age-related changes in auditory function are complex and can present heterogeneous audiometric patterns among individuals due to the cumulative effects of exogenous factors [2]. Nevertheless, our PTA results suggest similar audiometric patterns in hearing-impaired older adults, even after accounting for coexistent metabolic diseases. Moreover, it was observed that cochlear dysfunction affected both groups (i.e., NHL and HL) in the encoding of high-frequency information, contributing to the classic pattern of high-frequency hearing loss associated with aging [3], even in older adults with normal PTAs.

As expected, hearing-impaired older adults have a higher prevalence of tinnitus compared to their normal-hearing peers. However, even in the NHL group, approximately 32.3% reported tinnitus. These results are consistent with the higher-frequency PTA of this group, suggesting that tinnitus is associated with higher hearing thresholds. Furthermore, these results align with previous studies indicating that 60 to 80% of older adults above 70 years develop a hearing loss that primarily affects the basal end of the basilar membrane [3,25,26], leading to preservation of low-frequency hearing thresholds and detriment of high-frequency hearing. In fact, a sharp decrease in higher frequencies hearing thresholds may predispose the occurrence of tinnitus due to an abrupt discontinuity in the activity along the tonotopic axis [25,27,28].

Another complaint analyzed was dizziness, which showed a slightly higher prevalence in hearing-impaired participants. However, descriptions of episodes of dizziness were typical of benign paroxysmal positional vertigo [29], and therefore not necessarily related to ARHL [17,29]. BPPV is frequently associated with aging; however, ARHL has been more commonly linked to other inner ear pathologies, such as Ménière’s disease [30,31]. However, the interaction between auditory and vestibular systems, as well as cognitive and perceptual resources involved in postural control, suggests a multifactorial relationship between hearing loss and balance-related outcomes.

In our cognitive results, a general decrease in cognitive performance (i.e., MMSE total score) was associated with worse hearing in older adults with lower educational attainment [32,33]. In contrast, among individuals with higher education, this association was not observed. This pattern may reflect the contribution of cognitive reserve, whereby higher education is positively associated with the level of cognitive function and, therefore, provides improved coping abilities, potentially delaying the negative impact of hearing loss on cognitive performance [34]. Previously, Chen and Lu (2020) reported similar findings, with education acting as a moderating factor in this relationship and being considered a proxy for cognitive reserve, as years of formal education have been associated with the development of more efficient and flexible cognitive networks [35].

A possible explanation for these findings is that older adults with hearing loss and lower cognitive reserve may experience less neural efficiency with increasing task demands. This neural inefficiency may be associated with increased listening effort and, consequently, more extensive stimulus processing at the expense of other cognitive processes, such as working memory. In particular, this may limit resources available for attentional control and contextual updating processes required for orientation tasks. Therefore, more resource-demanding cognitive processes would reflect less flexibility and adaptability, overall resulting in poorer performance.

Our results suggest that the orientation domain is particularly sensitive to hearing loss, likely reflecting its reliance on the integration of multiple sensory inputs, with performance being influenced by altered attentional engagement in individuals with age-related hearing loss. Hence, degraded auditory input may lead to inefficient or insufficient engagement of attentional networks, particularly in older adults with lower educational attainment and reduced cognitive reserve. Consistent with the cognitive load hypothesis, increased effort in auditory processing may reduce the resources available for attentional control and other executive functions. As a result, long-term deficits in auditory function may reduce access to environmental and speech sounds, thereby impairing both temporal and spatial awareness and ultimately resulting in lower orientation scores. These findings are consistent with previous studies, such as Diao et al. (2021), who also reported significant associations between hearing thresholds and orientation performance [36].

With respect to language abilities, they were negatively associated with high-frequency PTAs, which may be partly explained by reduced speech intelligibility associated with high-frequency hearing loss. In line with our findings, Golub et al. (2020) reported a significant association between hearing loss and these cognitive domains using the Digit Symbol Substitution Test (DSST), a non-verbal cognitive measure, in a large sample of more than 5000 participants [32]. These results support the observed link between auditory and cognitive decline, rather than a test-related artifact.

Additionally, our results identified an association between low-frequency PTAs and the recall domain. One possible explanation is that reduced sensitivity to low frequencies may affect speech prosody and temporal cues, which are crucial for accurate speech encoding and subsequent recall [37,38]. Moreover, low-frequency PTAs may increase listening effort, thereby depleting cognitive resources available for memory consolidation and recall.

Consistent with our hypothesis, individual differences may counteract the impact of hearing loss on orientation and other cognitive domains, with those having higher reserve performing better, whereas individuals with lower reserve are more vulnerable to its detrimental effects [34,35].

This highlights the potential for higher educational attainment to counteract these detrimental age-related effects on the brain, possibly through mechanisms of cognitive reserve. Finally, interventions targeting auditory function, such as hearing aid fitting or auditory training programs, may help attenuate the impact of hearing loss on cognitive performance, especially in individuals with lower cognitive reserve.

### Study Limitations

A key limitation of our findings was the use of the MMSE as a screening tool for cognitive function. As a verbal tool, the MMSE may be influenced by hearing ability, specifically in the language and recall domain, which means that instructions can be difficult for hearing-impaired participants to perceive, potentially leading to an underestimation of accurate cognitive performance. Although cognitive assessments were conducted in a quiet room by a single trained examiner and only participants with mild to moderate hearing loss were included, the potential impact of hearing loss on test performance cannot be fully excluded. Furthermore, while the MMSE is a widely standardized neuropsychological tool, more comprehensive cognitive assessments may be required. Future work should therefore combine standardized scales with clinical interviews and behavioral observations, and particularly in ARHL, include non-verbal cognitive measures to minimize auditory bias. Another important limitation concerns the baseline difference in educational attainment between the NHL and HL groups. Although education was specifically examined as a moderating factor in the relationship between hearing and cognitive performance, the initial imbalance in years of education may have influenced overall group comparisons. For this reason, findings may benefit from interpretation within education-stratified analyses rather than simple between-group comparisons. While stratified analyses were conducted to mitigate this effect and to allow appropriate interpretation of MMSE scores according to educational attainment, residual confounding cannot be entirely excluded.

## 5. Conclusions

Our findings showed a significant association between hearing thresholds and cognitive performance in hearing-impaired older adults, supporting previous evidence that links auditory function to cognitive decline. Moreover, these effects were moderated by educational attainment. Domains such as orientation, language, and memory appear particularly vulnerable, likely reflecting the increased demands on attentional processing and consequent cognitive overload, which further supports the consideration of ARHL as a potentially modifiable risk factor for age-related cognitive impairment.

These findings highlight the importance of implementing auditory interventions, such as hearing aid fitting and auditory training programs, which may help mitigate the adverse effects of hearing loss on cognitive functioning, particularly in individuals with lower cognitive reserve.

## Figures and Tables

**Table 1:** Demographic characteristics by group.

	NHL (n = 31)	HL (n = 46)	*p* Value
Age (mean, SD)	71.4 (4.56)	73.1 (5.89)	0.18
Sex: male/female (n)	18/13	23/23	0.60
Education: years (mean, SD)	11.1 (5.45)	7.7 (4.56)	0.004 **
Diabetes: no/yes (n)	21/10	36/10	0.24
Hypertension: no/yes (n)	13/18	17/29	0.88
Hyperlipidemia: no/yes (n)	17/14	21/25	0.58

Abbreviations: HL, hearing loss; n, number; NHL, normal-hearing listener; SD, standard deviation. ** *p* < 0.01.

**Table 2:** Clinical characteristics by group.

	NHL (n = 31)	HL (n = 46)	*p* Value
Hearing level: PTA 0.5–4 kHz (dB) (mean, SD)	18.15 (4.96)	40.61 (8.35)	<0.001 ***
Low-frequency PTAs (dB) (mean, SD)	14.50 (4.75)	27 (8.88)	<0.001 ***
High-frequency PTAs (dB) (mean, SD)	29.22 (11.90)	58.67 (11.38)	<0.001 ***
Dizziness: no/yes (n)	26/5	36/10	0.77
Tinnitus: no/yes (n)	21/10	16/30	<0.01 **

Abbreviations: dB, decibel; HL, hearing loss; n, number; NHL, normal-hearing listener; PTA, pure-tone average; SD, standard deviation. ** *p* < 0.01; *** *p* < 0.001.

**Table 3:** Results of cognitive screening using the MMSE.

	NHL (n = 31)		HL (n = 46)	
	Lower Education	Higher Education	Lower Education	Higher Education
Orientation (mean, SD)	9.86 (0.53)	9.40 (0.74)	9.30 (0.95)	9.31 (1.03)
Registration (mean, SD)	3 (0)	3 (0)	3 (0)	3 (0)
Attention (mean, SD)	4.50 (0.65)	4.93 (0.26)	4.18 (1.40)	4.69 (0.63)
Recall (mean, SD)	2.21 (0.70)	2.40 (0.83)	1.94 (0.79)	2.23 (0.60)
Language (mean, SD)	7.36 (0.63)	7.87 (0.35)	7.03 (1.05)	7.92 (0.28)
Visuospatial (mean, SD)	0.71 (0.47)	0.93 (0.26)	0.58 (0.50)	0.85 (0.38)
MMSE total score (mean, SD)	27.64 (1.45)	28.69 (1.30)	26.28 (3.20)	28 (1.83)

Abbreviations: HL, hearing loss; n, number; MMSE, Mini-Mental State Examination; NHL, normal-hearing listener, SD, standard deviation.

## Data Availability

The datasets presented in this article are not readily available because they contain sensitive personal information and are therefore not publicly accessible. Requests to access the datasets should be directed to the corresponding author upon reasonable request.

## Abbreviations

The following abbreviations are used in this manuscript:

ARHL	Age-related hearing loss
DSST	Digit Symbol Substitution Test
HL	Hearing loss
MMSE	Mini-Mental State Examination
NHL	Normal-hearing listener
PTA	Pure-tone average
SNHL	Sensorineural hearing loss
WHO	World Health Organization
